# The Effects of Lithium Chloride Exposure on the Reproduction of *Caenorhabditis elegans*

**DOI:** 10.17912/micropub.biology.000988

**Published:** 2024-02-01

**Authors:** George Hatzis, Olivia Rossi, Izabella Testiler, Grace Dobbins, Erica Homan

**Affiliations:** 1 Department of Biology, Northeastern University, Boston, Massachusetts, United States

## Abstract

*Caenorhabditis elegans* ( *C. elegans)* are model organisms that share similar anatomical structures to humans. By exploring the effects of lithium chloride (LiCl) on *C. elegans,* we can collect crucial data regarding the compound’s impact on patients taking psychiatric medications containing LiCl. Here we performed an egg retention assay on nematode populations to explore how LiCl can influence reproduction. We found a statistically significant difference in eggs retained between control and experimental groups, suggesting that LiCl has negative effects on reproductive health.

**Figure 1. The average number of eggs retained by C. elegans exposed to LiCl is significantly less than the control:**
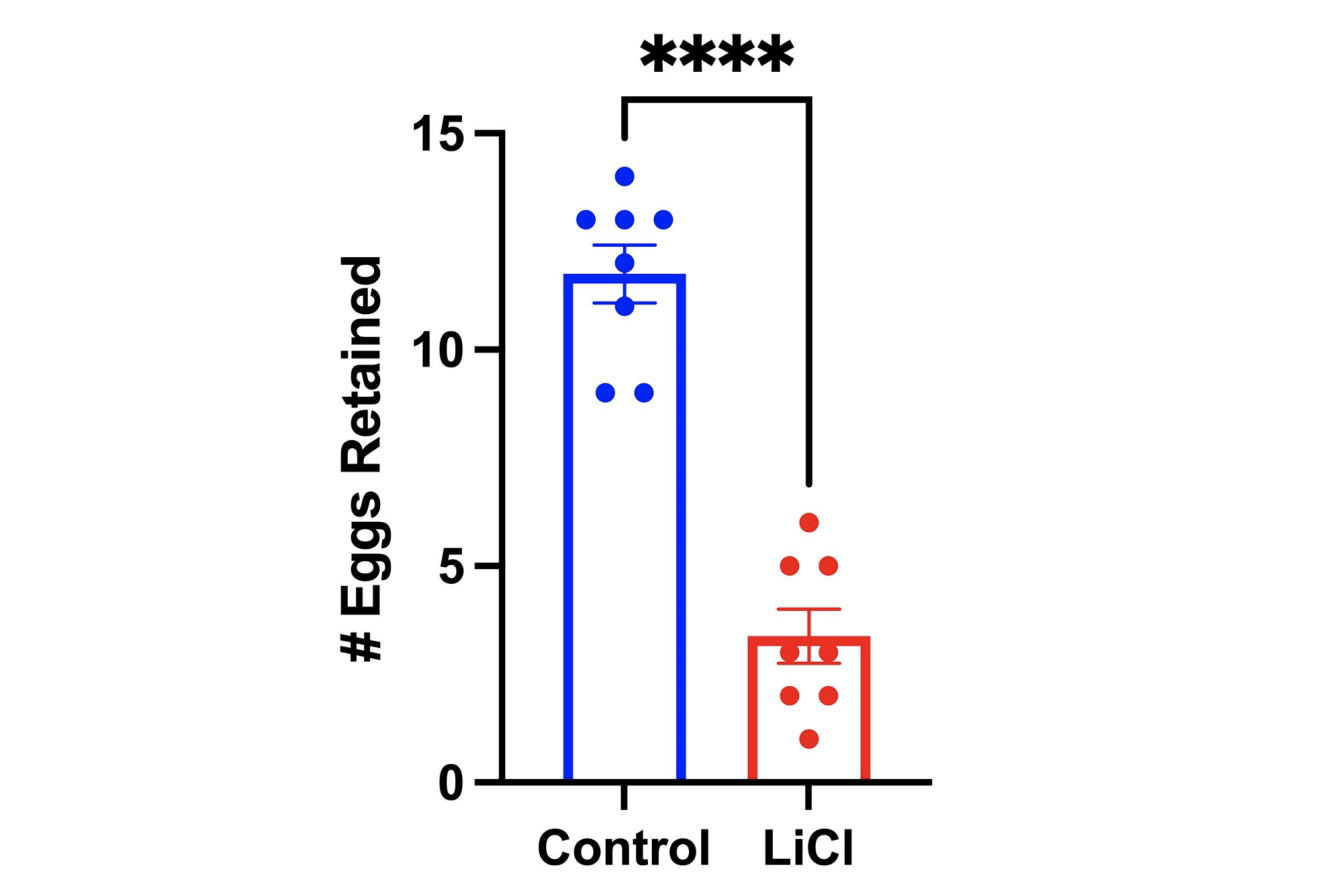
Fifteen to twenty L4 stage nematodes were transferred to 10cm plates with or without 10mM LiCl for 48 hours and incubated at 25°C. Nematodes from each plate were observed to count the number of eggs retained with three independent trials per group, where the graph plots the results of 1 of the 3 trials. The dependent variable is the number of eggs retained, the independent variable is the presence or absence of 10mM LiCl, and the control variables are the number of nematodes used for each condition, the storage environment for the nematodes, and the 48-hour time frame over which the worms were exposed to LiCl. The average number of eggs retained in each type of growth condition is shown in the bar plot (n=8, p < 0.0001). A t-test was also performed to determine the significant difference between the number of eggs retained between both groups (n=8, p < 0.0001) where **** represents a p-value < 0.0001. Additionally, a 2-way ANOVA was performed, which showed no difference between the trials, but a difference between treatments. The error bars represented are of the standard error of the mean (SEM).

## Description

LiCl is a chemical compound used in laboratories for various experimental purposes and is also an FDA-approved medication for psychiatric conditions such as bipolar disorder and major depressive disorder (Dols et al., 2013) . The industrial use of LiCl, via the use of lithium mines and lithium collection pools has made the environmental and ecological impacts of lithium a concern, raising questions about the efficacy of this compound in modern medicine (Dols et al., 2013) . Even at low concentrations, LiCl can lead to lithium poisoning and side effects in the neurological, gastrointestinal, endocrine, and metabolic systems (Dols et al., 2013) . Prior research conducted to better understand the therapeutic mechanisms suggests that exposure of *Caenorhabditis elegans* ( *C. elegans* ) to LiCl can serve as a model to study the effects of the chemical on a wide array of biological functions, which may be applicable to human populations too (McColl et al., 2008) .

*C. elegans* is a small nematode – roundworm – that is found in warm soils, vegetation, and compost, where the organism can feed on bacteria and fungi (Schulenburg & Félix, 2017). *C. elegans* is a model organism in laboratories due to its simple biological anatomy, short genome, rapid ~ 14-day lifecycle, and the fact that it is transparent (Riddle et al., 1997) . These characteristics allow users to easily grow nematodes and study the effects of external variables on the properties of the organism, like lifespan, motility, mobility, and more (Riddle et al., 1997) . Given that *C. elegans* and humans share a prehistoric common ancestor, their anatomical compositions are quite similar (Marsh & May, 2012) . Due to this parallel, *C. elegans* research can form the basis for studies in other animals that may eventually lead to clinical studies in human populations. This investigation will utilize the aforementioned model organism to explore how lithium chloride (LiCl) affects the reproductive capabilities of *C. elegans* .

*C. elegans* are primarily found as hermaphrodites that are capable of reproducing approximately 3 days after birth once they finish spermatogenesis, switch to oogenesis, and develop into adults (Riddle et al., 1997) . In general, a hermaphrodite has between 10-15 eggs in the uterus at a given time (Gardner et al., 2013) . Our experiment attempts to explore how LiCl exposure affects *C. elegans’* reproduction by carrying out egg retention assays on experimental groups containing LiCl and control groups without LiCl. We hypothesized that when *C. elegans* were exposed to LiCl, the nematodes’ reproduction, seen via eggs retained, will decrease.

48 hours after starting the experiment, the control group of nematodes not exposed to LiCl retained 11.1 eggs; this value falls within the expected range of 10 – 15 eggs that are found within the uterus of mature *C. elegans* ( Figure 1 ). In contrast to the control group, there was a significant 56.8% decrease in eggs retained to an average of 4.8 eggs in the treatment group exposed to LiCl ( Figure 1 ). Three independent trials further confirmed our results. The data collected from the egg retention assay supports the argument that LiCl exposure negatively affects *C. elegans’* reproduction. When exposed to LiCl, the number of eggs in the nematodes was over two times smaller than the control group exposed to just E. coli OP50 , supporting the reproduction argument of our hypothesis.

Prior literature also supports these conclusions. A study focused on the effects of lithium exposure on various parameters of rat sperm, such as the rate of spermatogenesis and sperm quality, found that exposing rodents to LiCl in varying doses leads to decreases in the total number of sperm, sperm motility, morphology, and the rate at which sperm were produced. This suggests negative reproductive effects associated with the chemical, where a concentration of 30mg/kg of body weight had the most detrimental impact (Dashti et al., 2013) . The fact that *C. elegans* is a model organism sharing common ancestry with rodents and humans, implies that the conclusions of the aforementioned study have a degree of translatability to the research conducted in our experiment. These parallels show that different organisms can experience similar effects when exposed to lithium and help further validate that the findings of this study are not due to external influences or variables. Due to the parallels between *C. elegans* and humans, our findings on the effect of LiCl can set the scene for future studies that may explore the effects of the chemical on human populations. LiCl is a widely approved FDA medication for psychiatric disorders, however, there are speculations regarding the safety and efficiency of the drug (Dols et al., 2013) . Our data show that LiCl is detrimental to reproductive processes in the nematode, and a closer look could uncover further effects on animal health. While generalizations to patients cannot be immediately made, these conclusions suggest that new restrictions may have to be implemented to ensure clinical safety.

Future investigations should start by performing additional reproduction and egg retention assays to better understand the effects of LiCl on nematodes; these could include brood size assays and egg laying assays to better quantify areas of reproduction and fertility that are affected by LiCl. Various concentrations of LiCl ranging from high to low should be incorporated into replicate trials to explore whether different concentrations of the drug have adverse effects on egg retention, which can be visualized via a dose-response curve. The assay should also be replicated on rodent populations and non-human primates which share more biological similarities with humans. This will gradually increase the translatability of data relating to fertility and reproduction, which can eventually lead to clinical trials that explore the effects of LiCl on human populations. In the meantime, investigating how LiCl affects the rate at which nematodes lay eggs, the number of eggs laid over a worm's lifetime, and the variations in maturity levels reached by LiCl-exposed nematodes versus control groups, all present feasible experiments to better understand the detrimental effects of this chemical on fertility and reproductive health.

Overall, the findings from our egg retention assay support the hypothesis that LiCl negatively impacts reproduction in *C. elegans* and suggests that the chemical is harmful to nematodes. However, additional research on the biological effects of lithium is needed to comprehensively understand how nematodes are affected, to identify novel ways of testing these effects on other animal populations, and to inevitably pursue clinical research that may reveal similar detrimental effects associated with lithium-based drugs in patient populations.

## Methods

Egg Retention Assay: 15 – 20 L4 *C. elegans* nematodes were transferred and maintained on the center of OP50 -seeded NGM agar plates. Plates were split into two groups with three plates in each; one group contained 10mM LiCl mixed into the plate, whereas the other did not. The plates were then incubated at 25°C for 48 hours. Eight 10μl drops of bleach were placed on the lid of a 10cm petri dish, and one nematode was then transferred into each of the bleach drops in order to dissolve the outer cuticle. Nematodes were left in the bleach for 10 minutes. Each dissolved nematode was visually observed with an Olympus SZ51 Stereo Zoom dissecting microscope at 10x magnification to count the number of eggs present, and egg retention data was averaged and graphed using the GraphPad Software. Three independent trials were conducted per group, where we plotted the results of 1 of the 3 trials. We also performed a 2-way ANOVA which showed no difference between the trials, but a difference between treatments. Lastly, a 2-sample t-test was used to calculate the p-value.

N2 *C. elegans* Maintenance: Wild-type N2 *C. elegans* were obtained from the Caenorhabditis Genetics Center (CGC) and were maintained on NGM plates seeded with OP50 .

## Reagents

- NGM Medium

- Lithium Chloride powder, Lot #: MKCF4863, Manufacturer: Sigma-Aldrich

- LB broth

- OP50

- N2 *C. elegans,* Caenorhabditis Genetics Center (CGC)

- Sodium Hypochlorite (Bleach)
